# Proteomic and metabolomic insights into oxidative stress response activation in mouse embryos generated by *in vitro* fertilization

**DOI:** 10.1093/hropen/hoaf022

**Published:** 2025-04-28

**Authors:** Seok Hee Lee, Saúl Lira-Albarrán, Paolo F Rinaudo

**Affiliations:** Department of Obstetrics and Gynecology, Center for Reproductive Sciences, University of California San Francisco, San Francisco, CA, USA; Department of Obstetrics and Gynecology, Center for Reproductive Sciences, University of California San Francisco, San Francisco, CA, USA; Department of Obstetrics and Gynecology, Center for Reproductive Sciences, University of California San Francisco, San Francisco, CA, USA

**Keywords:** embryo development, IVF, metabolomics, proteomics, oxygen concentration

## Abstract

**STUDY QUESTION:**

How different is the global proteomic and metabolic profile of mouse blastocysts generated by IVF, cultured in optimal (5% O_2_) or stressful (20% O_2_) conditions, compared to *in vivo* generated blastocysts?

**SUMMARY ANSWER:**

We found that in IVF-generated embryos: (i) the proteome was more sensitive to high oxygen levels than the global metabolomic profile; (ii) enzymes involved in splicing and the spliceosome are altered; (iii) numerous metabolic pathways, particularly amino acids metabolism, are altered (iv) there is activation of the integrated stress response (ISR) and downregulation of mTOR pathways.

**WHAT IS KNOWN ALREADY:**

IVF culture conditions are known to affect the gene expression of embryos. However, comprehensive data on the global metabolic and proteomic changes that occur in IVF-generated embryos are unknown.

**STUDY DESIGN, SIZE, DURATION:**

Mouse embryos were generated by natural mating (*in vivo* control or flushed blastocyst-FB-group) or by IVF using KSOM medium and two distinct oxygen concentrations: 5% O_2_ (optimal) and 20% O_2_ (stressful). Proteomic and metabolomic analyses were performed using state-of-the-art mass spectrometry techniques in triplicate (n = 100 blastocysts per replicate), allowing for detailed profiling of protein and metabolite alterations in each group.

**PARTICIPANTS/MATERIALS, SETTING, METHODS:**

Mouse blastocysts were collected from CD-1 and B6D2F1 strains as specified above. High-resolution liquid chromatography-tandem mass spectrometry (LC-MS/MS) was used for proteomics, while high-performance liquid chromatography coupled with mass spectrometry (HILIC-MS) was used for metabolomics. In addition, Immunofluorescence was used to assess the activation of stress response pathways, including the ISR.

**MAIN RESULTS AND THE ROLE OF CHANCE:**

Proteomic analysis revealed significant changes in protein expression in embryos cultured under 20% O_2_ compared to 5% O_2_ and *in vivo* embryos. Compared to *in vivo* embryos, IVF embryos cultured under 20% O_2_ exhibited 599 differentially expressed proteins, with an increase in proteins involved in oxidative stress responses, aminoacyl-tRNA synthesis, and spliceosome pathways. In contrast, IVF embryos cultured under 5% O_2_ showed fewer changes, with 426 differentially expressed proteins, though still reflecting significant alterations compared to *in vivo* embryos. These results indicate that embryos in stressful conditions (20% O_2_) exhibit a stronger stress response and alterations in critical pathways for protein synthesis and DNA repair. Metabolomic analysis revealed that embryos cultured under 20% O_2_ showed changes in branch-chained amino acid levels, and decreased levels of key metabolites of the TCA cycle and pentose phosphate pathway. Embryos cultured under 5% O_2_ had increased pyruvate levels, suggesting altered glycolysis. Immunofluorescence confirmed that oxidative stress markers such as GCN2, EIF2α, and ATF4 were upregulated in IVF embryos, indicating ISR activation. Overall, IVF and embryo culture have a direct impact on embryo proteomes and metabolomes affecting amino acid metabolism and stress-related pathways.

**LARGE SCALE DATA:**

N/A.

**LIMITATIONS, REASONS FOR CAUTION:**

Results in a murine model should be extrapolated with caution to human embryos.

**WIDER IMPLICATIONS OF THE FINDINGS:**

These findings offer valuable insights into how different IVF culture conditions, specifically oxygen levels, impact the global metabolic and proteomic profiles of embryos. These findings provide critical insights into the profound impact of IVF culture conditions, particularly oxygen levels, on the global metabolic and proteomic landscapes of embryos. By identifying key metabolic pathways disrupted by oxidative stress, we highlight the potential clinical importance of proteomic and metabolomic analyses in understanding embryo quality, improving ART, and ultimately enhancing pregnancy outcomes. The integration of metabolomic and proteomic data offers a comprehensive understanding of how oxidative stress influences cellular function. These insights have direct clinical relevance, providing a foundation for optimizing ART protocols to mitigate oxidative stress.

**STUDY FUNDING/COMPETING INTEREST(S):**

This work was supported by grant R01 HD108166-01A1 from the National Institute of Child Health and Human Development (NICHD) to P.F.R. The authors declare that there is no conflict of interest that could be perceived as prejudicing the impartiality of the research reported.

WHAT DOES THIS MEAN FOR PATIENTS? This study investigates how laboratory conditions used during IVF can affect the health and development of mouse preimplantation embryos. IVF is an essential treatment for couples struggling with infertility, but the conditions under which embryos are cultured, such as oxygen levels, may influence embryo growth and long-term health. In particular, embryos grown in stressful conditions, such as higher oxygen levels, may undergo changes in important processes, including cell signaling and metabolism, which could affect their development.

Our research specifically examines how changes in oxygen exposure of IVF-generated embryos affect their global protein and metabolic fingerprints with embryos generated by mating as the controls. We found that embryos cultured in high oxygen environments show changes in metabolic and protein pathways. These alterations may impact the embryos' ability to develop properly and may influence the long-term health of children born through IVF.

This study highlights the importance of optimizing laboratory conditions to reduce stress on embryos. By improving these conditions, such as by reducing oxygen levels, doctors may be able to increase the success rates of IVF and improve the health outcomes for children conceived through these methods. The findings are particularly relevant as the number of IVF procedures continues to rise globally, and improving embryo culture conditions could provide significant benefits for patients undergoing assisted reproductive treatments.

## Introduction

Embryo culture is an integral component of ARTs. Assisted reproductive technologies like IVF have been transformative in addressing infertility and have resulted in the birth of millions of children worldwide (Herbert *et al.*, 2023). While ARTs have shown remarkable success, concerns have been raised regarding their potential implications for the health and development of the offspring. Although the majority of ART offspring are healthy, several studies have indicated possible associations between ART and altered growth patterns (Ceelen *et al.*, 2009), heightened susceptibility to cardiometabolic dysfunction (Guo *et al.*, 2017), anomalous blood pressure patterns (Cui *et al.*, 2021), perturbed lipid metabolism (Guo *et al.*, 2017), and even elevated susceptibility to chronic diseases in childhood (Knoester *et al.*, 2008). It is likely that epigenetic changes occurring in preimplantation embryos are responsible for these health changes. However, it is also essential to consider that factors other than the medium used to culture the embryos, including the type of fertilization, length of embryo culture (Bruna de Lima *et al.*, 2023), as well as modifications of the endometrial environment secondary to superovulation (Harvey *et al.*, 2025), could also contribute to these effects. The exact nature of molecular mechanisms following embryo culture and driving possible epigenetic changes are unknown. It is therefore of paramount importance to understand how preimplantation embryos react to the culture environment. We and others have shown that the culture of mouse embryos in different media profoundly affects the gene expression of the embryos. In particular, exposure of embryos to atmospheric as opposed to physiologic oxygen (20% O_2_ vs 5% O_2_) results in additional and profound changes in gene expression. Further, embryos cultured in different media or oxygen concentrations display changes in fetal/placental growth and in postnatal growth, with a predisposition to glucose intolerance.

Importantly, preimplantation embryos show significant changes in their metabolism (Gardner and Harvey, 2015; Sharpley *et al.*, 2021). Cleavage-stage embryos primarily utilize pyruvate and lactate as energy sources, mainly metabolized via oxidative phosphorylation (OXPHOS), while morula and blastocysts, in addition to OXPHOS, show increased glucose utilization and glycolysis (Chronopoulou and Harper, 2015). Additionally, there is respiratory surge exhibited by blastocysts and principally in trophectodermal cells (Leese, 2012).

Although prior studies have evaluated the effects of selected intracellular metabolite changes on embryo development (Sharpley *et al.*, 2021), an unsupervised and global evaluation of metabolic and proteomic changes could provide a more complete understanding of pre-implantation embryo development. Recently, we performed a limited proteomic analysis and found that changes in glycolytic, pentose phosphate pathways (PPP), and tricarboxylic acid (TCA or citric acid) proteins were present in embryos cultured *in vitro* suggesting an increased Warburg metabolism in IVF-generated mouse embryos (Lee *et al.*, 2022). The increased Warburg metabolism observed in IVF-generated mouse blastocysts confirms earlier studies in which the proportion of glucose converted to lactate was found to dramatically increase in cultured blastocysts compared to *in vivo*-derived blastocysts (Gardner and Leese, 1990). Furthermore, these effects were corrected by the addition of amino acids and vitamins (Lane and Gardner, 1998). Here, we expanded the proteomic analysis by exploring all pathways that are changed following *in vitro* culture and added a comprehensive metabolomic analysis.

Therefore, the current study aims to provide a comprehensive proteomic and metabolomic analysis of mouse blastocysts generated: (i) by mating and flushed out of the uterus (*in vivo* development or flushed blastocysts—FB group, serving as the control), (ii) IVF under optimal conditions (IVF_5%O2_ group, cultured under 5% oxygen concentration), and (iii) IVF under stressful conditions (IVF_20%O2_ group, cultured under 20% oxygen concentration). In addition, we confirmed the results by analyzing the expression of key regulatory proteins, by immunofluorescence (IF).

By performing these experiments, we tested the hypothesis that oxidative stress imposed by hyperoxia (20% O_2_ vs 5% O_2_), elicits multiple responses in blastocysts. Furthermore, we explored a secondary hypothesis proposing that blastocysts have developed repair and resistance mechanisms, rendering them resilient to oxidative and metabolic stresses.

We found that embryos generated *in vitro* show significant changes in proteins and metabolism, confirming increased oxidative stress and activation of the integrated stress response (ISR) (Chen *et al.*, 2023; Woo *et al.*, 2024).

## Materials and methods

### Embryo collection, IVF, and embryo culture

Animal experiments were approved by the Institutional Animal Care and Use Committee of the University of California, San Francisco (Approval No.: AN195972-01D), and all animals were maintained according to the institutional regulation under a 12-h light/dark cycle with *ad libitum* access to water and food. IVF was performed as previously described (Nagy, 2003) using outbred mice (Donjacour *et al.*, 2014). In brief, 5 IU pregnant mare serum gonadotrophin (PMSG; Mybiosource Inc., San Diego, CA, USA) was injected into CF1 female mice (8–9 weeks), and 48 h later, 5 IU human chorionic gonadotropin (hCG; Sigma-Aldrich Inc., Saint Louis, MO, USA) was injected into those female mice for superovulation. Sperm were collected from the cauda epididymis in B6D2F1 male mice (8–9 weeks), and cumulus–oocyte complexes (COCs) were obtained from ampullae 13–14 h after hCG administration. The COCs were incubated in a human tubal fluid (HTF) medium (Millipore Corp., Billerica, MA, USA) with an appropriate concentration of sperm obtained from 1-h capacitation at 37°C in a 5% CO_2,_ 5% O_2_ in humidified air. Four hours post-fertilization, the embryos were washed with several drops of potassium simplex optimization medium (KSOM; Millipore Corp.) and cultured in the same medium to the blastocyst stage at 37°C under LifeGuard Oil (CooperSurgical., Trumbull, CT, USA). The zygotes were cultured under two different conditions: (i) 37°C, 5% CO_2_ in humidified air, 5% oxygen (IVF_5%O2_ group), and (ii) 37°C, 5% CO_2_ in humidified air, 20% oxygen (IVF_20%02_ group). Embryo development was assessed based on morphological criteria, with key developmental milestones occurring at ∼16–18 h post-IVF for the 2-cell stage and 108–110 h post-hCG injection for the blastocyst stage. Control embryos (FB) were obtained from flushed blastocysts. Briefly, CF1 female mice (8–9 weeks) were superovulated with 5 IU PMSG and, 48 h later, 5 IU hCG. Then, females were mated to B6D2F1 males. The vaginal plug was checked 14–18 h post-hCG, and *in vivo*-generated blastocysts were isolated by flushing 90–96 h post-hCG. For all experiments, only expanded blastocysts with an intact ICM and a well-organized TE layer were selected for experiments.

To understand the dynamic proteomic and metabolic remodeling during embryo development, we used mass spectrometry (MS)-based proteomics and metabolomics to directly measure protein and metabolite abundance in embryos. In the case of metabolomics, due to difficulties in obtaining many blastocysts and the limited detection range of current metabolomics technologies, firstly, we optimized a targeted metabolomics approach to detect metabolites using a small number of blastocysts.

**Figure 1.**
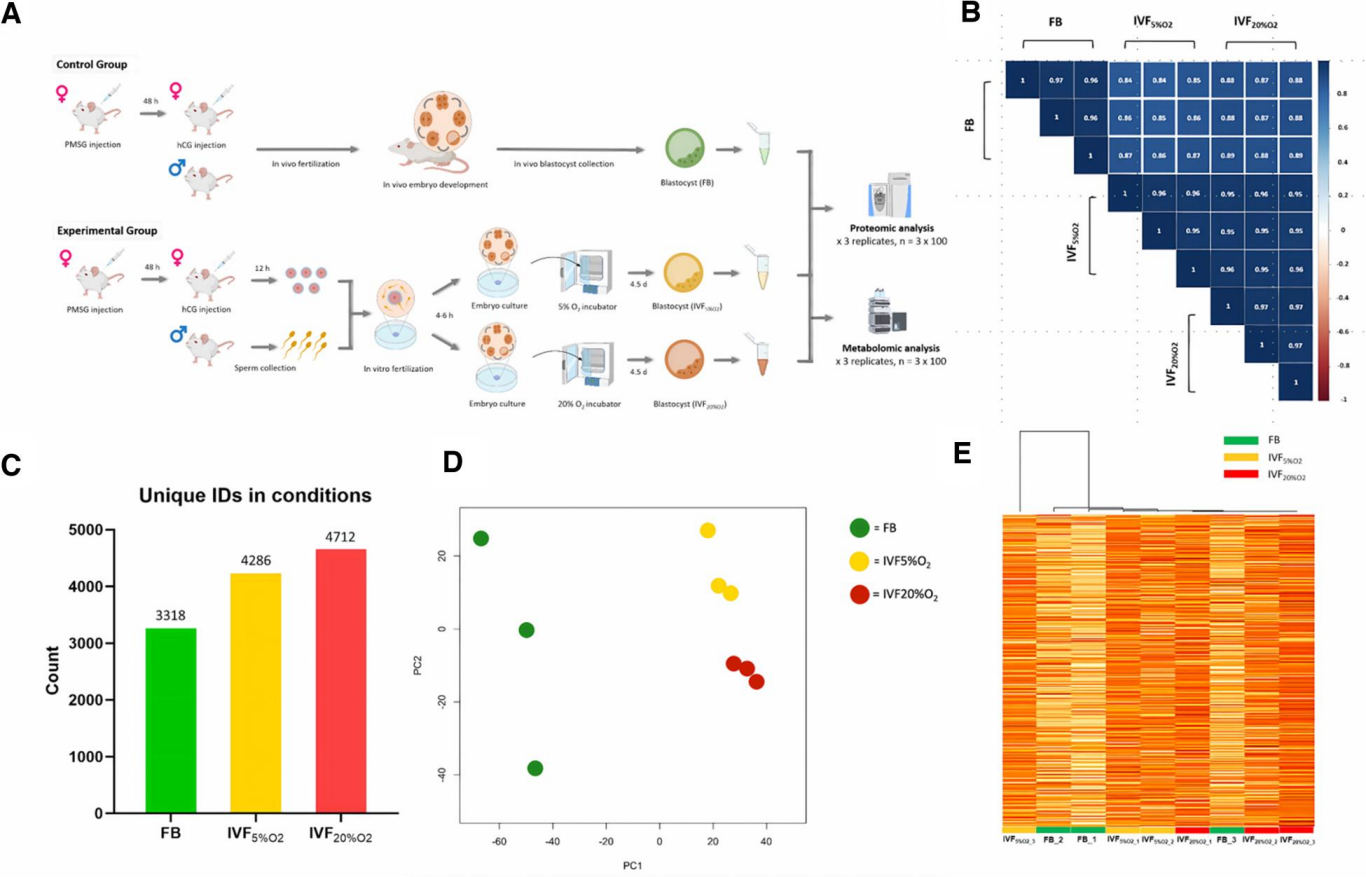
**Proteomics profiling of blastocyst-stage embryos derived from *in vivo* and *in vitro*.** (**A**) Schematic workflow for sample processing and targeted proteomics and metabolomics profiling of embryos. The processes depicted in *in vivo/in vitro* embryo development illustrate the stages of embryo development, starting from the zygote stage following IVF and progressing to the blastocyst stage. (**B**) Quality control of proteomic analysis. The figure presents pairwise correlations between the samples within each group and across different groups. The values inside the blue squares represent the correlation coefficients (*r*-values) between the proteomic profiles of the samples, where a value closer to 1 indicates a higher degree of similarity and reproducibility between the samples. The intensity of the blue color corresponds to the strength of the correlation, with darker blue indicating higher correlation and white or lighter colors indicating lower correlation. The color bar on the right provides a visual reference for the correlation values, ranging from low (red) to high (blue). (**C**) Number of unique protein identifiers across the three experimental conditions. (**D**) PCA plot for targeted proteomics analysis shows that biological replicates clustered based on the methods of fertilization and culture. Biological replicates of *in vivo* embryos (FB group) are green, whereas IVF embryos cultured under 5% O_2_ (IVF_5%O2_) and 20% O_2_ (IVF_20%O2_) are yellow and are red, respectively. The PCA plot shows the distribution of samples along the first two principal components (PC1 and PC2), which capture the most variance in the data. (**E**) Unsupervised hierarchical clustering heat map showing the expression levels of proteins across three experimental groups: FB, IVF_5%O2_, and IVF_20%O2_. Each group includes three biological replicates. The color intensity of each row represents absolute quantification (raw data), with higher intensity (red) indicating higher expression. Green, yellow, and red lines below each column represent the FB, IVF_5%O2_, and IVF_20%O2_ groups, respectively. Biological samples did not cluster based on the method of conception, indicating that while several proteins are different, the global changes are small. Statistical significance for differential protein expression was determined using one-way ANOVA followed by *post hoc* Tukey’s test (*P*<0.05). FB, *in vivo* fertilized embryos; IVF_5%O2_, *in vitro* fertilized embryos cultured under 5% oxygen; IVF_20%O2_, *in vitro* fertilized embryos cultured under 20% oxygen; PCA, principal component analysis; PC1, principal component 1; PC2, principal component 2.

We titrated a range of blastocysts from 25 to 100 blastocysts, and we were able to achieve strong correlations between the number of embryos and the MS signal for most of the metabolites even at lower input ranges of the gradients, indicating good quantification of those metabolites with this approach. Final experiments were conducted with 100 blastocysts per biological replicate and three biological replicates per group (n = 300 for FB, n = 300 for IVF_5%O2_, and n = 300 for IVF_20%02_). The same number of embryos (different collections) was used for proteomic analysis (Fig. 1A).

### Proteomic analyses

Experiments were conducted in triplicates (n = 100 blastocyst for each replicate). For proteomics analysis, the proteins were extracted with 8 M urea, 50 mM ammonium bicarbonate (Sigma-Aldrich), and benzonase 24U/100 ml (Sigma-Aldrich), reduced with TCEP (Thermo Fisher Scientific, Waltham, MA, USA), alkylated with iodoacetamide (Sigma-Aldrich), and digested overnight with Trypsin/Lys-C mix (Promega, Madison, WI, USA) followed by desalting with C18 cartridges in an automated fashion using an AssayMap BRAVO (Agilent Technologies, Santa Clara, CA, USA). The peptide mixture was analyzed by liquid chromatography with tandem MS (LC-MS/MS) using a Proxeon EASY nanoLC system (Thermo Fisher Scientific) coupled to an Orbitrap Fusion Lumos mass spectrometer equipped with FAIMS Pro device (Thermo Fisher Scientific).

Peptides were separated using an analytical C18 Aurora column (75 µm × 250 mm, 1.6 µm particles; IonOpticks, Fitzroy, Australia) at a flow rate of 300 nl/min using a 140-min gradient: 1–6% B in 1 min, 6–23% B in 90 min, 23–34% B in 48 min, and 34–50% B in 1 min (A=FA 0.1%; B = 80% ACN: 0.1% FA). The mass spectrometer was operated in positive data-dependent acquisition mode, and the Thermo FAIMS Pro device was set to standard resolution. A three-experiment method was set up where each experiment utilized a different FAIMS Pro compensation voltage: −50, −70, and −80 volts, and each of the three experiments had a 1-s cycle time. A high-resolution MS1 scan in the Orbitrap (m/z range 350–1500, 120k resolution at m/z 200, AGC 4e5 with maximum injection time of 50 ms, RF lens 30%) was collected in top speed mode with 1-s cycles for the survey and the MS/MS scans. For MS2 spectra, ions with charge state between +2 and +7 were isolated with the quadrupole mass filter using a 0.7 m/z isolation window, fragmented with higher-energy collisional dissociation (HCD) with normalized collision energy of 30%, and the resulting fragments were detected in the ion trap as rapid scan mode with AGC of 5e4 and maximum injection time of 35 ms. The dynamic exclusion was set to 20 s with a 10 ppm mass tolerance around the precursor.

All mass spectra were analyzed with SpectroMine software (Biognosys, Zürich, Switzerland, version 2.7.210226.47784). Search criteria used were full tryptic specificity (cleavage after lysine or arginine residues unless followed by proline), missed cleavages were allowed, carbamidomethylation (C) was set as fixed modification, and oxidation (M) as a variable modification. The false identification rate was set to 1%. Quality control (QC), relative quantification, and downstream analysis were performed using the artMS Bioconductor package, which uses MSstats for normalization and differential analysis (Choi *et al.*, 2014). Proteins were categorized as differentially expressed when absolute fold change >2 and adjusted *P*-value <0.05.

### Metabolomic analyses

Metabolomics analysis was conducted using hydrophilic interactions liquid chromatography (HILIC) coupled with MS (LC-MS) in both negative and positive ion modes. Optima grade LC-MS solvents for the MS analyses were purchased from Thermo Fisher Scientific. Individual isotopically labeled standards were purchased from Cambridge Isotope Laboratories (Tewksbury, MA, USA) and CDN Isotopes (Pointe-Claire, Quebec, Canada).

### Metabolomics sample preparation and analysis via hydrophilic interaction ion chromatography high-resolution tandem mass spectrometry (HILIC-HRMS/MS) analysis

A new set of frozen blastocyst samples (n = 900) were collected, with 300 embryos allocated to each experimental group. To ensure biological replication, each group was divided into three biological replicates, with 100 blastocysts per replicate. These embryos were obtained from different animals, ensuring biological variability across replicates. Samples were prepared as previously described (Mancini *et al.*, 2021). Briefly, blastocysts were lysed in 100 μl ice-cold lysis buffer (1:1:2, v: v: v, acetonitrile: methanol: 0.1 M ammonium bicarbonate—pH 8.0) containing isotopically labeled standards and sonicated in an ice bath for 30 min. Following lysis, proteins were precipitated by adding ice-cold methanol (800 μl) and incubated overnight at −80°C. Precipitated proteins were pelleted by centrifugation (7860 *g*, 15 min), and metabolite extracts were dried down *in vacuo*. Individual samples containing isotopically labeled standards were reconstituted in 40 μl of acetonitrile/water (80:20, v/v). A pooled QC sample was prepared by pooling equal volumes (10 μl) from each sample following reconstitution. Isotopically labeled standards were added during and after sample preparation to assess sample process variability and instrument variability.

Prepared samples were analyzed by HILIC high-resolution tandem MS (HILIC-HRMS/MS) in the Vanderbilt Center for Innovative Technology (CIT) (Nashville, TN, USA) using a modified version of hydrophilic interaction ion chromatography in both positive and negative ionization methods (Popay *et al.*, 2021; Mouton *et al.*, 2023). Briefly, metabolites were separated on a Waters Corporation (Milford, MA) ACQUITY UPLC BEH Amide HILIC column (100 × 2.1 mm, 1.7 μm particle size) using a water/acetonitrile gradient with ammonium formate (5 mM) added to both mobile phases. HRMS/MS analyses were performed on a high-resolution Thermo Fisher Scientific Q-Exactive HF hybrid quadrupole-Orbitrap mass spectrometer (Bremen, Germany) equipped with a Thermo Fisher Scientific Vanquish UHPLC binary system and autosampler controlled by Xcalibur 4.4 software (Waltham, MA, USA). Liquid chromatography was performed at 200 μl min^−1^ using solvent A (5 mM ammonium formate in 90% water, 10% acetonitrile, and 0.1% formic acid) and solvent B (5 mM ammonium formate in 90% acetonitrile, 10% water, and 0.1% formic acid) as previously described (Mouton *et al.*, 2023).

Mass spectrometry (full MS) analyses and tandem (MS/MS) data (6 and 8 μl injection volume for positive and negative ion mode, respectively) were acquired as previously described (Paudel *et al.*, 2020; Sekhar *et al.*, 2023). Pooled QC samples were used for column conditioning (eight QC samples injected before individual sample analysis and every five sample injections), retention time alignment, and to assess MS instrument reproducibility throughout the sample batch. Individual samples were randomized and injected one time in the sample batch. Quality assurance (QA) practices were applied to assess the analytical method performance. Lastly, a system suitability sample (in-house mixture of amino acids and polar small molecules) was used to verify the stability of retention times, peak shapes, and areas before and after sample batch analysis.

### Metabolomics data processing and statistical analysis

Spectral features (retention time, m/z pairs) were imported, processed, and normalized (integration) using Progensis QI v. 3.0 (Non-linear Dynamics, Waters Corporation). All MS and MS/MS sample runs were aligned against a pooled QC sample reference run, and spectral features were de-adducted and de-isotoped. Data were further curated by applying QA practices to the data. Specifically, spectral features with >25% coefficient of variation (CV) in the pooled QC samples were removed (total: 2168 spectral features or compounds). Data were normalized to all spectral features using Progenesis QI.

Sample process and instrument variability were assessed to determine sample and batch acceptance. Briefly, QA metrics for sample process and instrument variability are ≤10% CV and ≤5% CV, respectively. No samples were identified as outliers in these studies based on QA/QC practices. Statistical analyses were performed in Progensis QI using variance-stabilized measurements achieved through log normalization to calculate *P*-values by one-way ANOVA test. Significantly changed metabolites were chosen with the criteria *P*-value <0.05.

Validated, tentative, and putative annotations were determined within Progenesis QI using accurate mass measurements (<5 ppm error), isotope distribution similarity, retention time, and or fragmentation spectrum matching database searches against the Human Metabolome Database (HMDB) (Wishart *et al.*, 2013), METLIN (Smith *et al.*, 2005), and the CIT’s in-house library. Annotations (Levels 1–3) (Schrimpe-Rutledge *et al.*, 2016) were assigned for all significant compounds (*P*-value <0.05) with a match to any of the searched libraries or databases, specified in the subsequent section.

### Functional enrichment analysis

For proteomics data, the functional enrichment analysis was performed using g: Profiler^b^ (version e109_eg56_p17_1d3191d) with g: SCS multiple testing correction method applying significance threshold of *P*-adjusted value <0.05 (Raudvere *et al.*, 2019), loading the list of differentially expressed proteins. The overrepresented biological processes (BPs) and molecular functions (MFs) were summarized with REVIGO (reduce+visualize Gene Ontology, http://revigo.irb.hr) to avoid redundant gene ontology terms (Supek *et al.*, 2011). The nomenclature of BPs and MFs uses the Gene Ontology Consortium (Ashburner *et al.*, 2000; Gene Ontology *et al.*, 2023). The enriched canonical pathways (CPs) defined by KEGG (Kanehisa and Goto, 2000), Reactome (Milacic *et al.*, 2024), and WikiPathways (Martens *et al.*, 2021) were also identified with g: Profiler^b^. For metabolomic profiles, the enriched metabolite sets (EMS) were identified with MetaboAnalyst 5.0 with a false discovery rate (FDR) <0.05 (Pang *et al.*, 2022).

The proteomic and metabolomics profiles were evaluated with Ingenuity Pathway Analysis (QIAGEN IPA, http://digitalinsights.qiagen.com/IPA). The statistically significant CPs [−log(*P*-value) >1.3, using the right-tailed Fisher’s exact test], as well as the bio-functions (*P*-value <10E-3), were defined by the QIAGEN Knowledge Base. The absolute *Z*-score value that identifies the activation state was also evaluated (Kramer *et al.*, 2014). Furthermore, based on a hypothesis-driven approach of IVF culture conditions on metabolomics profiles, a molecule activity predictor (MAP) analysis by IPA was done as previously described (Lira-Albarran *et al.*, 2017, 2019).

### Immunocytochemical analysis

IF staining was performed to evaluate the pathway for the ISR in blastocysts derived from each group. Blastocysts were washed three times in PBS (Fisher Scientific, Hanover Park, IL, USA) containing 0.2% PVA (MilliporeSigma, Burlington, MA, USA) and fixed with 4% paraformaldehyde (w/v) (Fisher Scientific) in PBS for 30 min at room temperature. All procedures were conducted at room temperature unless otherwise stated. After washing three times in PBS, blastocysts were permeated with 1% (v/v) Triton X‐100 (Fisher Scientific) in PBS for 2 h. Then, the samples were washed three times in PBS and blocked with 2% BSA (Sigma-Aldrich) in PBS for 4 h at 4°C. The blastocysts were incubated with primary antibodies against GCN2 (1:200; E1V9M, Cell Signaling, Danvers, MA, USA), PERK (1:200; C118320, LSBIO, Seattle, WA, USA), EIF2α (1:200; D7D3, Cell Signaling), phospho-EIF2α (1:200; D9G8, Cell Signaling), ATF4 (1:200; B3517, LSBIO), LARS (1:200; C290957, LSBIO), AARS (1:200; C833398, LSBIO), WARS (1:200; C334267, LSBIO), mTOR (1:200; C368624, LSBIO), and S6K1 (1:200; A10982, Antibodies Inc, Davis, CA, USA). Blastocysts were then washed three times in PBS with 2% BSA and then incubated with a secondary anti‐rabbit antibody (1:200, ab150077, Abcam, Cambridge, MA, USA; 1:200, ab150075, Abcam; 1:200, ab150078, Abcam; 1:200, ab150080, Abcam) for 3 h at room temperature. Following three washes in PBS with 2% BSA, the samples were mounted on glass slides using ProLong Gold Antifade Mountant (P36930, Thermo Fisher Scientific).

Images were captured under a Nikon scanning confocal microscope with the same exposure times and adjustments. The intensities of PERK, EIF2α, phospho-EIF2α, ATF4, LARS, AARS, WARS, mTOR, and S6K1 were measured by Image J software (version 1.46 r; National Institutes of Health). For the analysis of PERK, EIF2α, phospho-EIF2α, ATF4, LARS, AARS, WARS, mTOR, and S6K1, at least three independent experiments were performed.

### Statistical analyses

Experiments were performed in triplicates. All data were analyzed by the one-way ANOVA with Tukey’s multiple comparison test or unpaired *t*-test when indicated using GraphPad Prism 9.0 (GraphPad, San Diego, CA, USA). All data are expressed as the means values±SD. Differences were considered statistically significant if *P* < 0.05.

## Results

### Embryo development

To assess the effects of different oxygen concentrations on embryo development, we compared the first cleavage and blastocyst formation rates between the IVF_5%O2_ and IVF_20%O2_ groups. The first cleavage rate was significantly higher in the IVF_5%O2_ group (83.8 ± 5.1%) compared to the IVF_20%O2_ group (70.8 ± 4.0%, *P* < 0.0001). Similarly, the blastocyst formation rate was also higher in the IVF_5%O2_ group (88.0 ± 3.8%) than in the IVF_20%O2_ group (78.2 ± 3.6%, *P* < 0.0001).

### Proteomics of IVF group (IVF_5%O2_ and IVF_20%O2_) vs control group (flushed blastocysts)

QC analysis of proteomics data showed consistent and reproducible results within each group (Fig. 1B). A total of 3318 (FB), 4286 (IVF_5%O2_), and 4712 (IVF_20%O2_) unique proteins were identified in each group (Fig. 1C). Principal component analysis (PCA) showed that, as expected, samples clustered based on the methods of fertilization and culture (Fig. 1D). However, an unsupervised clustering heat map showed that samples did not cluster based on the method of fertilization, indicating that the changes were small (Fig. 1E).

**Figure 2.**
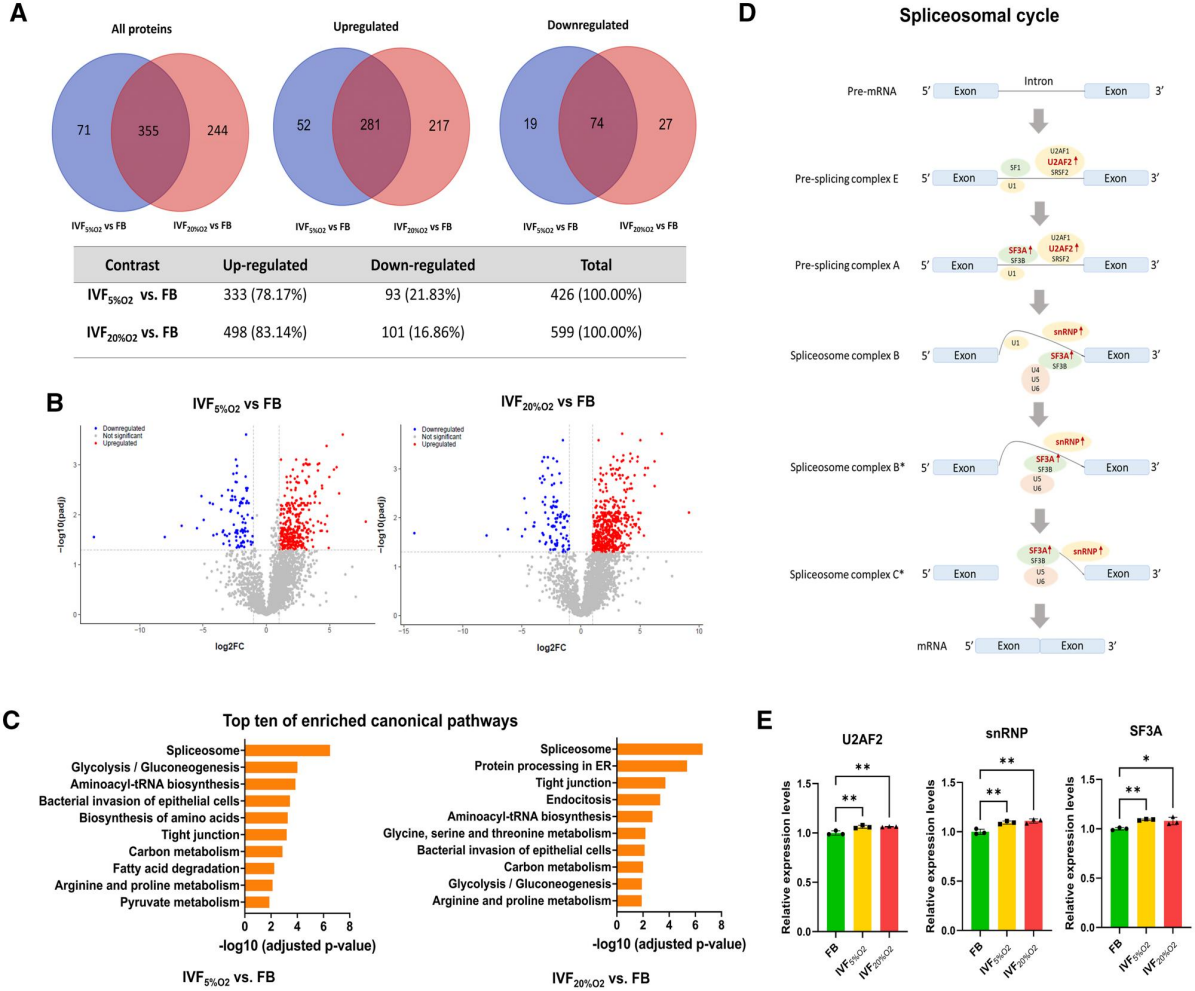
**Proteomics profiling of blastocyst-stage embryos derived from *in vivo* and *in vitro*.** (**A**) Venn diagram and accompanying table showing the overlap among up-regulated/down-regulated proteins between FB, IVF_5%O2_, and IVF_20%O2_ groups. (**B**) Volcano plots illustrating the differentially expressed proteins between FB and IVF groups. The *x*-axis represents the log_2_-fold change in gene expression between the groups, and the *y*-axis represents the −log_10_ of the adjusted *P*-value. Upregulated genes in the IVF group are shown in red and those downregulated are shown in blue. (**C**) Top 10 enriched KEGG canonical pathways identified in IVF group compared to the FB group ordered by statistical significance. (**D**) Schematic representation of the spliceosomal cycle, illustrating key steps including assembly, catalysis, and disassembly phases. Proteins highlighted in red indicate significant upregulation in the IVF group. (**E**) Protein expression levels of U2AF2, SF3A, and snRNP. The bar graph shows the relative expression levels of each protein in the IVF group compared to the FB group. Data are presented as mean±SD, with statistical significance determined by unpaired two-tailed Student’s *t*-test and indicated by asterisks: *P<0.05, **P<0.01. FB, *in vivo* fertilized embryos; IVF, *in vitro* fertilized embryos; IVF_5%O2_, *in vitro* fertilized embryos cultured under 5% oxygen; IVF_20%O2_, *in vitro* fertilized embryos cultured under 20% oxygen; snRNP, small nuclear ribonucleoprotein; U2AF2, U2 small nuclear RNA auxiliary factor 2; SF3A, splicing factor 3A subunit.

Embryos cultured in optimal conditions (IVF_5%O2_) showed 426 differentially expressed proteins (adjusted *P* < 0.05), 333 up-regulated (78.17%), and 93 down-regulated (21.83%) compared to *in vivo* flushed blastocysts (Fig. 2A and Supplementary File S1: volcano plot—Fig. 2B). Embryos cultured in stressful conditions (IVF_20%O2_ group) had 599 differentially expressed proteins (n = 498 up-regulated and n = 101 down-regulated) compared to *in vivo* embryos (Fig. 2A and Supplementary File S2). Overall, more proteins were up-regulated in IVF-generated embryos (nearly 80%), and IVF embryos shared a large proportion of different proteins compared to *in vivo* embryos (355/426 proteins = 83.33% IVF_5%O2_ and 355/599 = 59.27% in IVF_20%O2_ embryos, Fig. 2A and Supplementary File S1). In addition, 244 proteins differed in embryos cultured in low vs high oxygen (Fig. 2A).

Although extensive functional enrichment analysis for gene ontology terms and CPs was performed for comparison IVF_5%O2_ vs FB and IVF_20%O2_ vs FB (Supplementary Files S1 and S2), the most succinct and relevant results derived from CPs defined by KEGG (Fig. 2C). When comparing the top 10 CPs changed in embryos cultured *in vitro* (both IVF_5%O2_ and IVF_20%O2_)_,_ an abundance of pathways belonged to metabolism (6/10 pathways in IVF_5%O2_ vs FB comparison and 4/10 pathways in IVF_20%O2_ vs FB). In addition, the spliceosome pathway, aminoacyl-tRNA synthesis, and tight junction were changed in all IVF embryos (Fig. 2C–E and Supplementary Files S1 and S2). Protein processing in the endoplasmic reticulum and endocytosis pathways are uniquely changed in embryos cultured in high oxygen (Fig. 2C).

Aminoacyl-tRNA synthesis emerged as one of the most significant pathways altered in IVF-generated embryos with eight aminoacyl-tRNA synthetases (TARS, GARS, LARS, EPRS, MARS1, AARS, WARS, and VARS) up-regulated in IVF embryos (Table 1).

**Table 1.** Different expression levels of aminoacyl-tRNA synthetase proteins in *in vitro* fertilized embryos compared to the control *in vivo* embryos.

Protein name	IVF_5%O2_		IVF_20%O2_	
	Fold change	Adjusted *P*-value	Fold change	Adjusted *P*-value
WARS	5.36	0.006	4.21	0.011
TARS	4.64	0.007	4.08	0.008
AARS	3.79	0.019	2.67	0.036
MARS1	3.68	0.009	3.62	0.008
LARS	2.83	0.033	2.95	0.036
GARS	2.74	0.031	2.09	0.049
EPRS	2.15	0.0007	2.86	0.0003
VARS	2.14	0.014	2.11	0.012

TARS, threonyl-tRNA synthetase; GARS, glycyl-tRNA synthetase; LARS, leucyl-tRNA synthetase; EPRS, glutamyl-prolyl-tRNA synthetase; MARS1, methionine-tRNA synthetase 1; AARS, alanyl-tRNA synthetase; WARS, tryptophanyl-tRNA synthetase; VARS, valyl-tRNA synthetase.

Several critical spliceosome proteins (27 proteins out of 157), 14 of which in all IVF embryos were significantly dysregulated in IVF embryos (Table 2), including U2 small nuclear ribonucleoprotein auxiliary factor 2 (U2AF2), splicing factor 3a (SF3A), and small nuclear ribonucleoprotein (snRNP) (Fig. 2D and E).

**Table 2.** Different expression levels of proteins involved in the spliceosomal cycle in IVF-generated embryos compared to the control group.

Protein name	IVF_5%O2_		IVF_20%O2_	
	Fold change	Adjusted *P*-value	Fold change	Adjusted *P*-value
WBP11	7.87	0.019	14.92	0.008
RBM17	4.98	0.029	8.00	0.011
SRSF5	4.36	0.050	4.68	0.040
SRSF3	3.84	0.032	3.77	0.003
SF3A1	3.72	0.028	3.19	0.035
SNRPD2	3.31	0.006	2.89	0.012
PRPF6	3.31	0.041	4.72	0.015
SMNDC1	3.29	0.028	4.1	0.012
SNRNP40	3.29	0.026	3.88	0.011
SNRNP200	2.83	0.040	2.47	0.036
U2AF2	2.34	0.025	2.29	0.018
HNRNPC	2.29	0.025	2.27	0.021
SRSF1	2.12	0.027	2.09	0.030
ALYREF	2.10	0.010	2.72	0.006

WBP11, WW domain binding protein 11; RBM17, RNA binding motif protein 17; SRSF5, serine and arginine-rich splicing factor 5; SRSF3, serine and arginine-rich splicing factor 3; SF3A1, splicing factor 3a; subunit 1; SNRPD2, small nuclear ribonucleoprotein D2; PRPF6, pre-mRNA splicing factor 6; SMNDC1, survival motor neuron domain containing 1; SNRNP40, small nuclear ribonucleoprotein 40 (U5); SNRNP200, small nuclear ribonucleoprotein 200 (U5); U2AF2, U2 small nuclear ribonucleoprotein auxiliary factor (U2AF) 2; HNRNPC, heterogeneous nuclear ribonucleoprotein C; SRSF1, serine and arginine-rich splicing factor 1; ALYREF, Aly/REF export factor.

Overall, 244 proteins differed in embryos cultured in low vs high oxygen (Supplementary File S3). Surprisingly, the functional enrichment analysis identified only one enriched CP defined by KEGG, and one by Wikipathways based on these proteins, whereas six pathways belonging to mRNA process and metabolism were identified using Reactome software (Supplementary File S3).

### Metabolomics of IVF group (IVF_5%O2_ and IVF_20%O2_) vs control group (flushed blastocysts)

**Figure 3.**
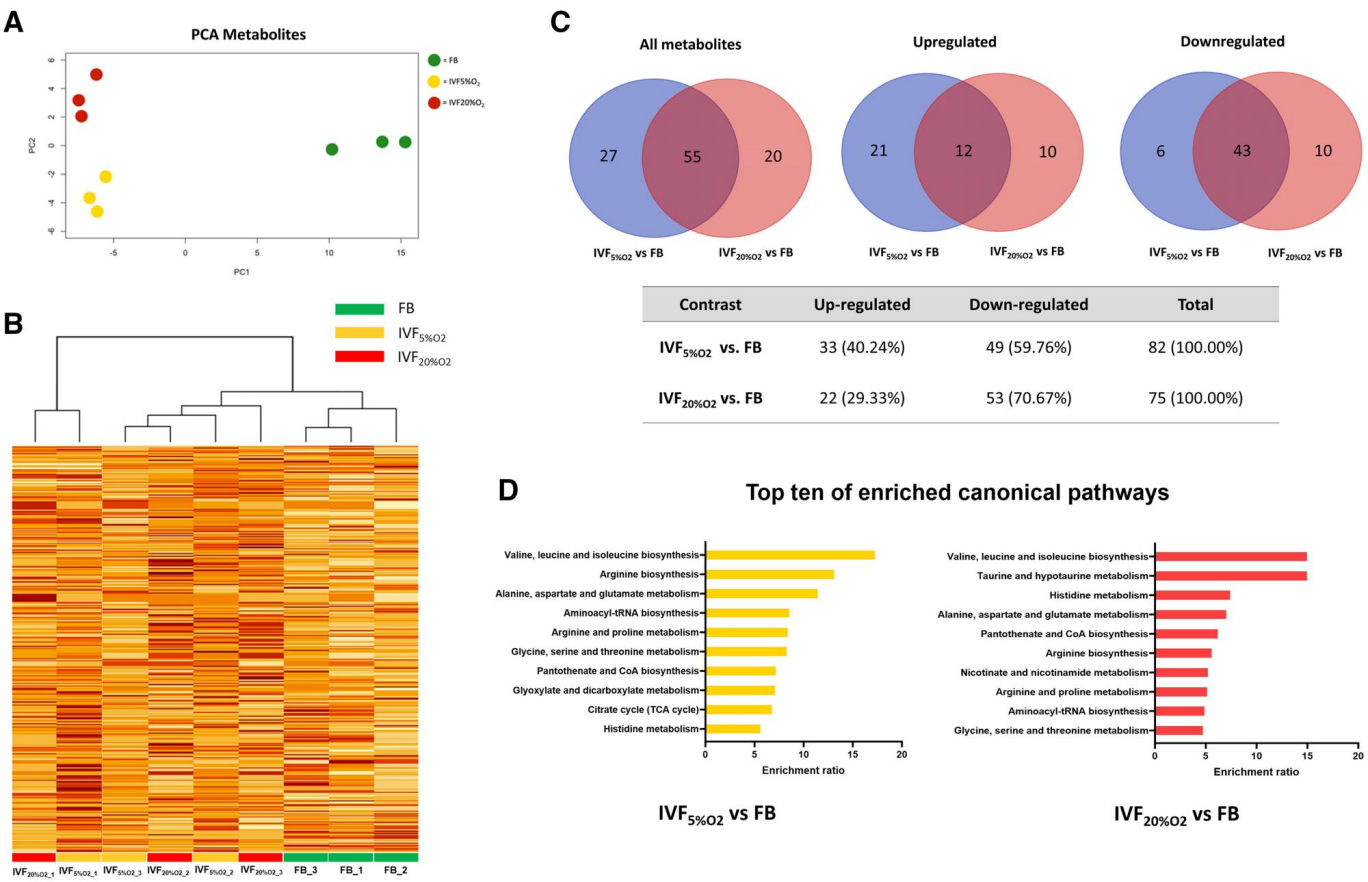
**Metabolomic profiling of mouse pre-implantation embryo.** (**A**) Unsupervised PCA reveals that samples cluster together based on the method of fertilization. Metabolites marked with the same color represent three biological replicates of the blastocyst-stage embryos. The PCA plot shows the distribution of samples along the first two principal components (PC1 and PC2), capturing the most variance in the data. (**B**) Unsupervised hierarchical clustering heat map showing expression levels across FB, IVF_5%O2_, and IVF_20%O2_ groups. Each group includes three biological replicates. The color intensity represents metabolite abundance (raw data), with higher intensity indicating higher expression. Green, yellow, and red lines below each column represent the FB, IVF_5%O2_, and IVF_20%O2_ groups, respectively. (**C**) Venn diagram showing the overlap of up-regulated/down-regulated metabolites among groups. (**D**) Top 10 enriched canonical pathways based on the Human Metabolome Database, as analyzed using MetaboAnalyst 5.0, were identified in each IVF group compared to *in vivo* embryos. Pathways are ordered by enrichment ratio. Statistical significance for differential metabolite expression was determined using one-way ANOVA followed by *post hoc* Tukey’s test (*P*<0.05). FB, *in vivo* fertilized embryos; IVF, *in vitro* fertilized embryos; IVF_5%O2_, *in vitro* fertilized embryos cultured under 5% oxygen; IVF_20%O2_, *in vitro* fertilized embryos cultured under 20% oxygen; PCA, principal component analysis; PC1, principal component 1; PC2, principal component 2.

Sample gradient titration (not shown) confirmed that 100 blastocysts per biological replicate were sufficient for reproducible and reliable metabolomic results. Using the HILIC positive and negative ionization modes, 2168 and 2289 metabolites were detected for IVF_5%O2_ and IVF_20%O2_, respectively. PCA revealed that, as observed with proteomics profiles, the IVF-generated embryos are more linked to themselves than the *in vivo* embryos (FB) (Fig. 3A).

Unsupervised clustering showed that *in vivo* embryos (FB group) but not IVF embryos, clustered together (Fig. 3B), indicating that IVF embryos had overall similar metabolic signatures and were different from *in vivo* conceived blastocysts.

Embryos cultured in optimal conditions (IVF_5%O2_ group) differed for 82 metabolites (*P* < 0.05) compared to those of *in vivo* embryos, with 49 of these metabolites (59.76%) showing lower levels, whereas 33 metabolites were found to be at higher levels (40.24%) in the IVF_5%O2_ group (Fig. 3C and Supplementary File S4). Embryos cultured in stressful conditions (IVF_20%O2_ group) differed for a similar number of metabolites to *in vivo* embryos: 75, of which 53 metabolites (70.67%) were at lower levels (Fig. 3C and Supplementary File S5). The embryos generated by IVF shared nearly 2/3 metabolites compared to *in vivo* embryos (55/82 = 67.07% for the IVF_5%O2_ group and 55/75 = 73.33% for the IVF_20%O2_ group). Notably, 9 out of the top 10 enriched CPs indicated metabolic alterations; the top enriched CP was the biosynthesis of branched-chain amino acids (BCAAs). Similar to the findings from the proteomic data, aminoacyl-tRNA biosynthesis is a significantly altered pathway (Fig. 3D). Additionally, 51 metabolites were differentially expressed between the IVF_20%O2_ and IVF_5%O2_ groups, with 12 up-regulated and 39 down-regulated in the IVF_20%O2_ group (Supplementary File S6). Eleven EMS were identified, with the highest enrichment observed in phenylalanine, tyrosine, and tryptophan biosynthesis, and aminoacyl-tRNA biosynthesis being a key finding, similar to the previous contrast analyses (Supplementary File S6). On the other hand, only two bio-functions, predicted by Ingenuity Knowledge Base, were activated in the IVF_20%O2_ group compared to the IVF_5%O2_ group: uptake of amino acids and transport of D-glucose (Supplementary File S6).

**Figure 4.**
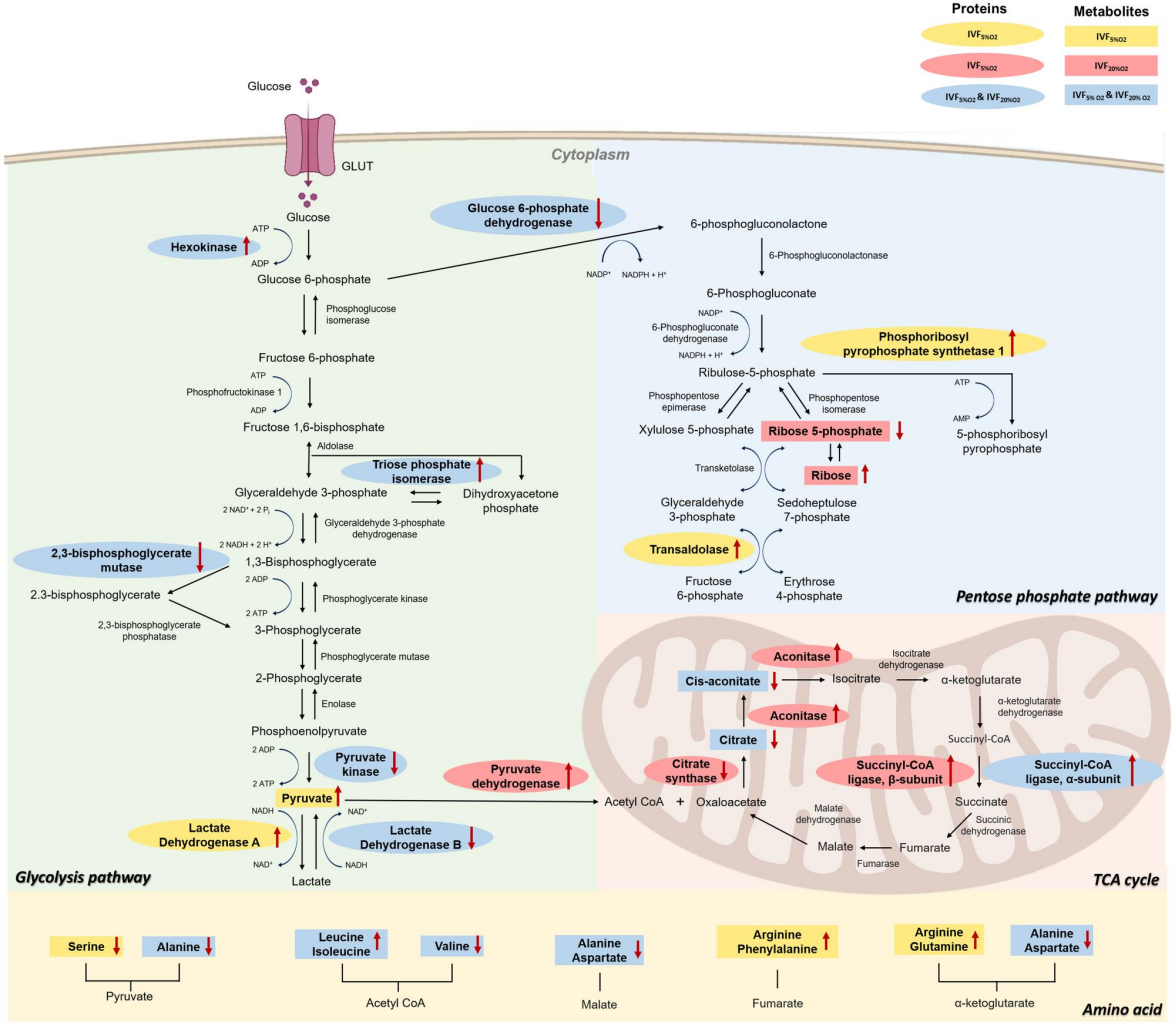
**Activities of metabolic pathways during mouse pre-implantation embryo development.** Schematic diagram illustrating the glycolysis, TCA cycle, and PPP, integrated with metabolomic and proteomic data. Metabolites are represented in rectangular shapes, whereas proteins are shown in oval shapes. Metabolites and proteins are highlighted in red color if they were statistically different (*P*<0.05) in both IVF groups compared to the control. Yellow color indicates changes observed only in IVF_5%O2_-generated blastocysts vs *in vivo* embryos, and blue color indicates changes of only in IVF_20%O2_-generated blastocysts vs *in vivo* embryos. Black arrows represent the directionality of the interactions. No significant differences were observed in the proteomic profiles between IVF_5%O2_ and IVF_20%O2_ blastocysts. Metabolomic analysis revealed that D-Ribose levels were higher in the IVF_20%O2_ group, whereas cis-aconitate was more abundant in the IVF_5%O2_ group. Arginine and leucine levels were significantly elevated in the IVF_5%O2_ group. Statistical analysis was performed using one-way ANOVA to assess significant differences in metabolite levels (*P*<0.05) between the IVF groups and *in vivo* embryos. IVF, *in vitro* fertilized embryos; IVF_5%O2_, *in vitro* fertilized embryos cultured under 5% oxygen; IVF_20%O2_, *in vitro* fertilized embryos cultured under 20% oxygen; TCA, tricarboxylic acid cycle; PPP, pentose phosphate pathway.

Among metabolites related to the glycolysis pathway, only pyruvate showed significantly higher levels in the IVF_5%O2_ blastocysts compared to the *in vivo* embryos (Fig. 4, Table 3 and Supplementary Fig. S1). In addition, two metabolites of the PPP [Ribose 5-phosphate (decreased) and ribose (increased)] were altered only in the IVF_20%O2_ group compared to the control, and two metabolites (cis-aconitate and citrate) of the TCA cycle decreased in both IVF groups compared to the control (Fig. 4, Table 3, and Supplementary Fig. S1).

**Table 3.** Proteins and metabolites (including amino acids) of glycolysis, pentose phosphate, and TCA cycles different between IVF-generated embryos compared to the control group.

	Protein		Metabolites	
	IVF_5%O2_	IVF_20%O2_	IVF_5%O2_	IVF_20%O2_
**Glycolysis**	Hexokinase		Pyruvate	–
	Triose phosphate isomerase			
	2,3-bisphosphoglycerate mutase			
	Pyruvate kinase			
	Lactate dehydrogenase A	–		
	Lactate dehydrogenase B			
**Pentose phosphate pathway**	Glucose 6-phosphate dehydrogenase			Ribose 5-phosphate
	Phosphoribosyl pyrophosphate synthetase 1	–	–	Ribose
	Transaldolase			
**TCA cycle**	–	Aconitase	Cis-aconitate	
		Pyruvate dehydrogenase		
		Succinyl-CoA ligase, β-subunit	Citrate	
		Citrate synthase		
	Succinyl-CoA ligase, α-subunit			
**Amino acids**	–		Serine	–
			Phenylalanine	
			Glutamine	
			Arginine	
			Leucine	
			Isoleucine	
			Alanine	
			Aspartate	
			Valine	

Several amino acids were significantly changed in IVF embryos compared to *in vivo* embryos (Fig. 4, Table 3, and Supplementary Fig. S1). BCAAs were changed in IVF embryos: valine was decreased, whereas leucine and isoleucine were increased. In addition, levels of aspartate, alanine, and valine were lower in both groups of IVF embryos. Four metabolites were changed only in IVF_5%O2_ embryos compared to the control group: glutamine, arginine, and phenylalanine were increased, while serine was decreased.

### Oxidative stress analysis

Given the known increased oxidative stress in IVF embryos (Kawamura *et al.*, 2010; Lee *et al.*, 2022), we further analyzed our data for evidence of alteration in these pathways. Although there were no antioxidant enzymes or metabolites different between the groups, eight metabolites (L-carnitine, taurine, choline, citrate, malonic acid, pyruvate, and L-arginine) related to the synthesis of reactive oxygen species (ROS), were different. Notably, there was a lower expression of metabolites associated with antioxidative activity, including L-carnitine, taurine, choline, and citrate in both IVF groups (Supplementary Fig. S2). Additionally, four metabolites related to lipid peroxidation (L-carnitine, taurine, L-phenylalanine, and L-arginine) were different. Taurine and L-carnitine, metabolites known to mitigate the process of lipid peroxidation, had significantly lower levels in the IVF group. L-phenylalanine and L-arginine were significantly higher in the IVF 5% group compared to both FB and IVF 20% groups (Supplementary Fig. S2).

### Activation of the ISR in IVF-generated embryos

**Figure 5.**
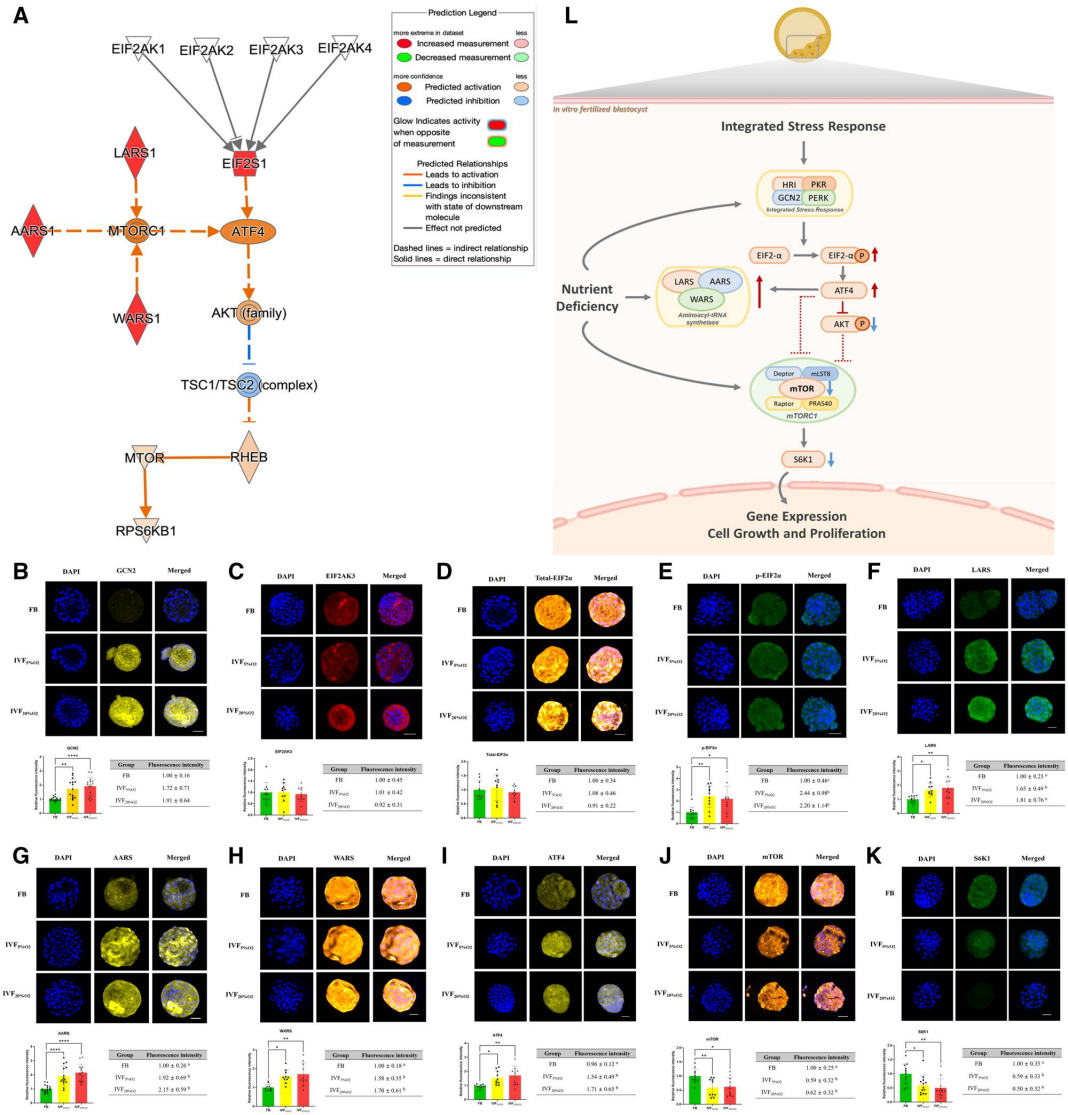
**IVF-generated embryos show activation of integrated Stress Response.** (**A**) Schematic illustration of the pathway involving GCN2 (EIF2AK4), EIF2, ATF4, mTOR, LARS, AARS, and WARS, generated using the MAP analysis by Ingenuity Pathway Analysis. The illustration highlights the predicted molecular interactions and regulatory relationships among these key proteins, providing insights into the activation and suppression patterns within the pathway. (**B–K**) Expression levels of essential proteins related to the ISR pathways in blastocysts from different conditions. ICC analysis of (**B**) GCN2 (48 blastocysts; Control: 18, 5% O_2_: 15, 20% O_2_: 15), (**C**) EIF2AK3 (PERK) (31 blastocysts; Control: 11, 5% O_2_: 10, 20% O_2_: 10), (**D**) Total-EIF2α (29 blastocysts; Control: 9, 5% O_2_: 10, 20% O_2_: 10), (**E**) *P*-EIF2α (33 blastocysts; Control: 11, 5% O_2_: 11, 20% O_2_: 11), (**F**) LARS (31 blastocysts; Control: 11, 5% O_2_: 10, 20% O_2_: 10), (**G**) AARS (48 blastocysts; Control: 18, 5% O_2_: 15, 20% O_2_: 15), (**H**) WARS (30 blastocysts; Control: 10, 5% O_2_: 10, 20% O_2_: 10), (**I**) ATF4 (30 blastocysts; Control: 10, 5% O_2_: 10, 20% O_2_: 10), (**J**) mTOR (32 blastocysts; Control: 11, 5% O_2_: 10, 20% O_2_: 11), and (**K**) S6K1 (37 blastocysts; Control: 12, 5% O_2_: 13, 20% O_2_: 12) were performed in blastocysts from three different groups. The scale bar in the figure represents a length of 50 µm. Statistical significance was assessed using one-way ANOVA followed by *post hoc* Tukey’s test. Data are presented as means±SD, with at least three independent replicates performed. Statistical significance is marked as follows: * *P* < 0.05; ** *P* < 0.01; (**L**) Illustration of activation of the ISR pathways in *in vitro* fertilized blastocysts. Nutrient deprivation secondary to culture conditions results in downregulation of mTOR and activation of ISR. The illustration highlights the relative changes in pathway activity in *in vitro* fertilized blastocysts (IVF) compared to *in vivo* embryos. IVF, *in vitro* fertilized embryos; IVF_5%O2_, *in vitro* fertilized embryos cultured under 5% oxygen; IVF_20%O2_, *in vitro* fertilized embryos cultured under 20% oxygen; GCN2, General Control Nonderepressible 2; EIF2AK3, Eukaryotic Translation Initiation Factor 2 Alpha Kinase 3; EIF2, Eukaryotic Translation Initiation Factor 2; ATF4, Activating Transcription Factor 4; mTOR, Mechanistic Target of Rapamycin; LARS, Leucyl-tRNA Synthetase; AARS, Alanyl-tRNA Synthetase; WARS, Tryptophanyl-tRNA Synthetase; ICC, Immunocytochemistry; p-EIF2α, Phosphorylated Eukaryotic Translation Initiation Factor 2 Alpha; S6K1, Ribosomal Protein S6 Kinase 1; ISR, Integrated Stress Response.

IPA predicted ATF4 and EIF2AK3 (a critical component of the ISR; Ryoo, 2024) as upstream regulators, whereas mTORC1 is predicted as the main downstream protein activated by LARS, EPRS, AARS, WARS, and VARS (Fig. 5A). Based on IPA prediction of activation of ISR and alteration in mTOR pathways and increase in selected aminoacyl tRNA synthetases (Fig. 5A), in IVF-generated embryos, we performed IF of selected proteins (GCN2, EIF2AK2, total-EIF2α, p-EIF2α, ATF4, LARS, AARS, WARS, mTOR, and S6K1) belonging to these pathways.

We found that except for EIF2AK3 (PERK) and total-EIF2α, which showed no significant difference between the FB and IVF groups, GCN2, p-EIF2α, ATF4, LARS, AARS, and WARS were significantly upregulated in the IVF-generated embryos (Fig. 5B–I) indicating activation of ISR response (Fig. 5L). Conversely, mTOR and S6K1 were significantly downregulated in the IVF embryos (Fig. 5J and K).

## Discussion

Our global proteome and metabolome analysis of mouse blastocysts generated *in vivo* or by IVF [either in optimal (IVF_5%O2_ group) or suboptimal conditions (IVF_20%O2_ group)] provide a trove of data that can help to understand how embryos adapt to the culture environment. We found that: (i) the proteome of embryos was more sensitive to high oxygen levels than the global metabolomic profile; (ii) alteration in enzymes involved in splicing and the spliceosome exist; (iii) numerous metabolic pathways, particularly amino acids metabolism, are altered following culture; and (iv) IVF-generated embryos show activation of ISR and downregulation of the mTOR pathway.

First, although all IVF-generated embryos showed similar proteomic and metabolic signatures (83% of protein and 67% of metabolites in IVF_5%O2_ were also changed in IVF_20%O2_), the proteome of embryos was more sensitive to high oxygen levels than the global metabolomic profile. The culture of embryos in atmospheric oxygen resulted in a higher number of dysregulated proteins (∼600) compared to the embryos in physiologic oxygen (426). Overall, 244 proteins, mainly involved in RNA metabolism, were changed in embryos cultured in high oxygen.

These findings align with previous studies demonstrating that exposure to atmospheric (20%) oxygen significantly influences preimplantation embryo development, metabolism, and gene expression. Gardner and Lane (2005) reviewed the profound negative effects of high oxygen tension on gene expression and imprinting patterns. Notably, exposure to atmospheric oxygen during the cleavage stages has been associated with increased amino acid utilization and altered carbohydrate metabolism (Wale and Gardner, 2012). Embryos cultured under 20% oxygen exhibit impaired ammonium regulation, a critical process for mitigating metabolic stress (Wale and Gardner, 2013). Furthermore, we found that mouse embryos cultured under 20% oxygen exhibit a 10-fold greater change in gene expression compared to those cultured under 5% oxygen (Rinaudo *et al.*, 2006). Additionally, embryos cultured at 20% oxygen show significant downregulation of multiple proteins compared to those cultured at 5% oxygen (Katz-Jaffe *et al.*, 2005). Finally, single embryos cultured under atmospheric oxygen experience metabolic stress and altered post-implantation development (Kelley and Gardner, 2019).

In contrast, a surprisingly similar number of metabolites changed in embryos cultured at 20% oxygen (n = 82 metabolites) and 5% oxygen (75 metabolites) when compared to *in vivo* embryos. This unexpected convergence in metabolite profiles implies that, at the metabolomic level, embryos respond comparably to both 20% and 5% oxygen conditions. The shared metabolic signatures suggest common adaptive strategies in response to the *in vitro* culture environment, irrespective of the specific oxygen concentration and a degree of metabolic robustness, implying that embryos may employ common metabolic strategies to navigate the challenges posed by *in vitro* culture in KSOM medium.

It is plausible that the metabolic pathways affected by oxygen concentration reach a saturation point or optimal response at 5% oxygen, leading to a plateau in metabolite alterations despite the subsequent increase in stress induced by 20% oxygen. Alternatively, embryos may activate similar compensatory mechanisms to cope with the *in vitro* conditions, regardless of the oxygen concentration, highlighting the resilience of early-stage embryos in adapting to their surroundings.

The extensive protein alterations under 20% oxygen hint at a more intricate and potentially stressful adaptive response, raising questions about the long-term consequences for embryonic development and viability.

The second important finding relates to changes in enzymes involved in splicing and the spliceosome. The spliceosomes, macromolecular ribonucleoprotein complexes with five core subunits and several cofactors, play a crucial role in the process of pre-mRNA splicing, a critical step in the maturation of messenger RNA (mRNA) molecules for protein synthesis (Wahl *et al.*, 2009; Will and Luhrmann, 2011). In this study, we identified several key spliceosome proteins, including U2AF2, SF3A, and snRNP, that exhibited increased expression levels in IVF embryos compared to *in vivo* embryos while no differences existed between the IVF groups (Fig. 2D and E). This finding is important since mutations in spliceosome complex proteins may lead to embryonic lethality before the blastocyst stage and developmental defects (Jablonka *et al.*, 2002; Fernandez *et al.*, 2018).

The increased levels of U2AF2, a key component involved in recognizing 3′ splice sites in pre-splicing complexes E and A (Zorio *et al.*, 1997), and of SF3A, a protein functioning in 3′ splice site recognition at early stages of spliceosome assembly (Tanackovic and Kramer, 2005), and elevated expression of snRNP proteins (increased in IVF embryos), crucial components of spliceosome complexes B, B*, and C*, suggest alterations in the early stages of spliceosomal assembly following embryo culture. Indeed, optimal functionality of the snRNP complex is essential for proper embryo development (Xiong *et al.*, 2022). Recent studies have shown that spliceosome inhibition can reprogram pluripotent stem cells into totipotent blastomere-like cells that form blastocyst-like structures and exhibit extended developmental potential (Lin *et al.*, 2021; Zhang *et al.*, 2023), highlighting the impact of *in vitro* conditions on embryonic development. A stress response due to the suboptimal condition compared to *in vivo* environments might elevate the expression of spliceosome proteins for compensatory mechanisms. In summary, these changes in spliceosomal proteins likely reflect the embryo’s attempt to fine-tune gene expression profiles critical for successful development outside the natural *in vivo* environment.

Next, we confirm that embryo culture is linked to increase in ROS; with culture under atmospheric oxygen exacerbating metabolic pathways associated with ROS production (Lee *et al.*, 2022; Jones *et al.*, 2023). Metabolomic data predicted an increase in the production, synthesis, and quantity of ROS and lipid peroxide (Supplementary Figs S1 and S2). Specifically, the significantly higher levels of cis-aconitate and citrate in FB-derived embryos compared to those derived from IVF suggest a more active TCA cycle in naturally conceived embryos (Supplementary Fig. S1). The TCA cycle plays a crucial role in cellular energy metabolism (Murphy and O’Neill, 2018; Hara *et al.*, 2021; Underhill and Cabeen, 2022), and its enhanced activity in FB embryos may indicate a greater metabolic efficiency in energy production. The reduced levels of these metabolites in IVF embryos could be linked to metabolic stress induced by *in vitro* culture conditions, potentially leading to alterations in mitochondrial function and overall embryonic development. Among amino acids, aspartic acid, alanine, and valine were significantly elevated in FB-derived embryos (Supplementary Fig. S1). Aspartic acid is a key intermediate in the malate–aspartate shuttle, which is essential for maintaining cellular redox balance and ATP production (Yang *et al.*, 2015; Ackermann *et al.*, 2023). Its reduced levels in IVF embryos may reflect alterations in mitochondrial NADH/NAD^+^ cycling, which could impact OXPHOS and cellular energy homeostasis. Alanine, which plays a role in gluconeogenesis and the glucose-alanine cycle (Perry *et al.*, 2018), was also lower in IVF embryos, suggesting possible disruptions in amino acid metabolism and energy substrate utilization under *in vitro* conditions. Additionally, valine, a BCAA critical for protein synthesis and mitochondrial function (Walejko *et al.*, 2021), was found in lower concentrations in IVF embryos, potentially implicating a reduced capacity for protein biosynthesis and metabolic adaptation.

It is notable that several key metabolites (L-carnitine, taurine, choline, and citrate, Supplementary Fig. S2) associated with ROS regulation had significantly higher levels in FB embryos. L-carnitine and taurine are essential for mitochondrial function and antioxidative defense, with L-carnitine facilitating fatty acid transport and β-oxidation (Longo *et al.*, 2016) and taurine contributing to cellular osmoregulation and mitochondrial stability (Jong *et al.*, 2021). Given their roles in mitochondrial function and antioxidative defense, L-carnitine and taurine are likely to contribute to enhanced oxidative stress resistance to FB embryos, potentially supporting improved metabolic efficiency and cellular homeostasis in our study. Choline plays a crucial role in phospholipid metabolism, contributing to membrane integrity and cellular signaling (Son *et al.*, 2024). The elevated choline levels observed in FB embryos may reflect enhanced phospholipid metabolism, potentially contributing to improved membrane stability and more efficient cellular communication. Furthermore, the increased levels of citrate, an important TCA cycle intermediate, could indicate enhanced metabolic flexibility (Li *et al.*, 2022b), allowing FB embryos to better manage oxidative stress and maintain homeostasis.

Additionally, factors related to lipid peroxidation, including L-carnitine and taurine, were significantly more abundant in FB embryos. Lipid peroxidation is a major consequence of oxidative stress, leading to membrane damage and impaired cellular function (Agmon *et al.*, 2018). The elevated levels of these metabolites in FB embryos suggest a more effective defense mechanism against lipid peroxidation, potentially contributing to improved membrane stability and cellular resilience under physiological conditions. Conversely, the lower levels of these protective metabolites in IVF embryos may render them more susceptible to oxidative stress-induced damage, which has been previously linked to compromised developmental potential in embryos generated *in vitro*.

Furthermore, ATF4 and EIF2AK3, two predicted transcription factors associated with an increase in amino-acyl tRNAs, are linked to ROS elevation (Chen *et al.*, 2019; Sun *et al.*, 2021; Zhu *et al.*, 2021).

Third, the *in vitro* culture of embryos induces changes in various proteins and metabolites crucial for essential metabolic pathways (Fig. 4 and Table 3). Among the notable alterations, amino acids stood out prominently. Five out of 20 amino acids exhibited statistically significant differences in embryos generated through IVF, with an additional four amino acids showing changes exclusively in embryos cultured under 5% oxygen conditions (Table 3).The significantly higher expression of those amino acids (arginine, leucine, isoleucine, phenylalanine) in IVF_5%O2_ derived embryos compared to both IVF_20%O2_ embryos and FB embryos suggests that embryos cultured under the optimal *in vitro* culture conditions (5% O_2_) may rely more heavily on these amino acids for metabolic processes. First, arginine is involved in critical functions such as energy production, protein synthesis, and the regulation of nitric oxide synthesis. The elevated levels in IVF_5%O2_ embryos could reflect an adaptive response to optimize energy metabolism and support embryonic development under more physiologically relevant oxygen levels. Similarly, leucine, isoleucine, and phenylalanine are essential BCAAs involved in energy production and protein synthesis. The higher levels of these BCAAs in IVF_5%O2_ embryos may reflect a shift toward increased protein synthesis and energy utilization, which could be advantageous in conditions with controlled oxidative stress. This adaptive response could help IVF_5%O2_ embryos maintain metabolic balance and support development under more optimal oxygen conditions. In contrast, IVF_20%O2_ embryos, exposed to higher oxygen levels, may activate different stress response pathways, possibly leading to altered amino acid metabolism in a way that differs from both IVF_5%O2_ and FB embryos. A recent study demonstrated that individual embryo culture in atmospheric oxygen exacerbates metabolic stress and negatively impacts post-implantation development (Kelley and Gardner, 2019). This aligns with our findings that culture conditions significantly influence metabolic pathways, particularly amino acid metabolism. An additional possibility to explain the variability in amino acid concentrations and the higher levels of arginine and leucine levels observed in IVF_5%O2_ groups is that different metabolites have different stability under different culture conditions (Tarahomi *et al.*, 2019). These authors found that storage and culture conditions influence the composition of human preimplantation embryo culture media, with leucine and arginine being particularly affected. Amino acid metabolism has long been recognized as a critical determinant of developmental potential, and several studies have provided valuable insights into the role of amino acid turnover in embryonic development. For example, amino acid turnover in human embryos following embryo freezing and thawing can predict developmental potential, with developmentally competent embryos being more metabolically quiescent than those that arrest (Stokes *et al.*, 2007). Additionally, the influence of culture media composition on amino acid turnover and embryo development has been well-documented in bovine embryos (Orsi and Leese, 2004). In agreement with these findings, we observed changes in the levels of numerous amino acids (Table 3) in IVF-generated blastocysts some of which (glutamine, arginine, and phenylalanine) were changed only in the IVF_5%O2_ group.

Interestingly, all branched amino acids (BCAs: valine, leucine, and isoleucine) were altered in all IVF-generated embryos. For example, valine, a key intermediate in the TCA cycle (Hara *et al.*, 2021), was decreased in IVF embryos. Previous studies have reported similar changes in valine levels during mouse embryo culture (Lamb and Leese, 1994), whereas porcine embryos displayed release of valine into the medium (Booth *et al.*, 2005). This is important, as BCAA are essential for protein synthesis, energy metabolism, and various physiological processes. Elevated BCAA levels have been associated with metabolic disorders, cardiovascular diseases, and insulin resistance, underscoring the significance of our findings (Zhang *et al.*, 2017; White *et al.*, 2021; Vanweert *et al.*, 2022).

These data are particularly relevant since we and others have found that mouse offspring conceived by IVF exhibit metabolic differences compared to naturally conceived offspring (Lee *et al.*, 2022; Lira-Albarran *et al.*, 2022). We can speculate that the different levels of BCA in early embryos could impact the insulin-related metabolic health of IVF-derived offspring.

The lower levels of alanine in IVF embryos are also noteworthy since alanine metabolism is closely linked to energy homeostasis. The glucose–alanine cycle is vital in exchanging carbon skeletons and energy substrates between different cells (Lu *et al.*, 2023). Lower levels of alanine might influence the balance between glycolysis and OXPHOS, potentially altering the availability of energy substrate of developing embryos.

Among the amino acids exhibiting changes exclusively in embryos cultured under 5% oxygen conditions, the decrease in serine levels is particularly intriguing. Serine plays a crucial role in nucleotide, protein, and phospholipid synthesis (Murtas *et al.*, 2020), as well as in one-carbon metabolism essential for DNA methylation and embryonic development (Zeng *et al.*, 2019). The observed decrease in serine metabolism may indicate a potential impact on DNA methylation dynamics during early embryogenesis.

The unexpected finding of more amino acids exhibiting statistically different levels in embryos cultured under an optimal oxygen concentration compared to stressful conditions raises intriguing questions. Although it is difficult to speculate on a possible reason for these differences, it is possible that each unique culture condition creates unique stress that will induce a media-specific response in the embryos. Indeed, mouse embryos cultured in different conditions show specific adaptation to each culture condition (de Lima *et al.*, 2020).

Given the differences in amino acid levels, it is non-surprising that the pathway aminoacyl-tRNA synthesis is enriched in metabolomic and proteomic data for all IVF-generated embryos. Changes in aminoacyl-tRNA synthesis may impact the accuracy and efficiency of protein translation, potentially leading to developmental abnormalities (Gupta *et al.*, 2023).

The metabolic results also expand our prior findings of alteration in proteins and metabolites involved in several key metabolic pathways. For example, regarding the glycolysis pathways, while six enzymes were found to be altered in all IVF-generated embryos (the only addition being LDHA, which was increased only in IVF_5%O2_ embryos), only one (pyruvate, increased in IVF_5%O2_ generated embryos) out of 10 metabolites of the glycolysis pathway exhibited changes. The reason for this divergence is unclear, but we can hypothesize that the observed changes in enzymatic levels might be adaptive, aiming to maintain a stable metabolic flow through glycolysis, thereby keeping metabolite levels constant.

Our analysis of the TCA cycle also revealed more changes in enzyme levels than metabolites in all IVF-generated embryos (Table 3). Decreased levels of cis-aconitate and citrate in all IVF-generated embryos are particularly relevant considering the central role of the TCA cycle in energy production and the metabolism of carbohydrates, fats, and amino acids (Martinez-Reyes and Chandel, 2020; Sanchez-Garcia *et al.*, 2021; Sun *et al.*, 2022).

Citrate’s involvement in the citrate shuttle, which transports acetyl-CoA for lipid synthesis (Izzo *et al.*, 2023), underscores the potential impact of reduced citrate levels on lipid metabolism critical for early embryonic development. The increased expression of aconitase, pyruvate dehydrogenase, and succinyl-CoA ligase in embryos cultured at 20% oxygen suggests an adaptive response to altered oxygen environments, possibly aimed at meeting increased energy demands. Conversely, decreased citrate synthase levels in embryos cultured at 20% oxygen may serve as a protective mechanism against oxidative stress induced by excessive mitochondrial activity. However, the overall impact of citrate synthase downregulation on ROS levels is multifaceted and warrants further investigation.

The changes observed in the PPP are complex. Glucose 6-phosphate dehydrogenase (G6PD), the first enzyme of the pathway responsible for generating NADPH, showed decreased levels in all IVF embryos, indicating altered cellular redox balance during *in vitro* development. Conversely, increased levels of phosphoribosyl pyrophosphate synthetase 1 and transaldolase in embryos cultured under 5% oxygen suggest an increased need for nucleotide production and carbon rearrangement in response to lower oxygen levels. Additionally, alterations in D-Ribose and D-Ribose-5P levels in embryos cultured at 20% oxygen suggest potential changes in RNA synthesis rates, contributing to observed significant changes in gene expression following embryo culture (Feuer *et al.*, 2016).

Based on the findings of this study, we propose a mechanism by which embryos respond to culture conditions (Fig. 5). Artificial culture conditions impose nutritional stress on the embryos, significantly impacting glycolysis, the PPP, the TCA cycle, and amino acid metabolism. Additionally, the upregulation of spliceosomal components may represent an adaptive response to fine-tune gene expression profiles to the culture conditions. This adaptive response helps maintain cellular homeostasis but also activates oxidative stress and the ISR. The ISR is a crucial cellular mechanism that cells utilize to adapt to various stressful conditions (Poncet *et al.*, 2020). In response to stress, such as nutrient deprivation or oxidative stress, the ISR activates various kinases, which phosphorylate EIF2α, leading to a reduction in global protein synthesis while selectively increasing the translation of specific stress-related proteins, such as ATF4 (Poncet *et al.*, 2020). Indeed, we found that GCN2, the kinase that play a pivotal role by sensing amino acid depletion is increased in IVF-generated embryos (Castilho *et al.*, 2014).

Our IF results confirm the predicted activation of ISR: GCN2, EIF2α, and ATF4 are increased in IVF-generated embryos. Furthermore, ATF4 enhances the synthesis of aminoacyl-tRNA synthetases, which are crucial substrates for amino acid transport (Krokowski *et al.*, 2013). Our findings corroborate a recent study that showed the loss of the amino acid transporter Slc7a5 in mouse embryos leads to rapid induction of the ISR (Poncet *et al.*, 2020).

Although ISR can be activated by multiple kinases and types of stress, amino acid deprivation is a common activator of EIF2α via the kinase GCN2. This is further supported by the lower activation of mTOR and S6K signaling in IVF-generated embryos. Notably, there are known interconnections between mTOR and ATF4. For example, the activation of ATF4 expression in cancer cells inhibits the AKT/mTOR signaling pathway, leading to cell cycle arrest and autophagy (Shao *et al.*, 2022). It is relevant that we had found reduced AKT phosphorylation at Ser473 in IVF-generated blastocysts (Liu *et al.*, 2010).

Additionally, we utilized KSOM as the culture medium, which contains L-glutamine as one of its key components. Although L-glutamine is essential for cellular metabolism, its breakdown during *in vitro* culture can lead to the production of ammonium, a byproduct known to be toxic to developing embryos (Wale and Gardner, 2013). It has been suggested that high oxygen concentrations can exacerbate oxidative stress, and when combined with the ammonium produced from glutamine breakdown, this may increase cellular stress levels in the developing embryos (Wale and Gardner, 2013). However, in our study, we also included sodium pyruvate, L-glutamic acid, L-alanine, and L-arginine in the medium, which helps to support the embryos’ metabolic needs and reduce the toxicity associated with ammonium accumulation (Tannen and Kunin, 1982; Goodman *et al.*, 1984; Khan and Yoshida, 2008; Li *et al.*, 2022a). These metabolites and nutrients can help buffer the potential harmful effects of ammonium, providing a metabolic alternative to minimize its impact on developing blastocysts. Despite the inclusion of these supportive components, we acknowledge that the 20% oxygen used in our culture system might have compounded the stress induced by ammonium. The high oxygen environment could promote the formation of ROS, which, in combination with the ammonium-induced toxicity, could further disrupt the delicate balance required for proper embryonic development.

Although our study provides valuable insights into the proteomic and metabolomic changes in IVF-generated embryos under varying oxygen concentrations, there are some limitations. Firstly, the study was conducted using a mouse model, which may not fully replicate the complexities of human embryo development. Additionally, although we studied how different oxygen concentrations affect embryo development in a single medium, other variables such as insemination conditions, different culture media, or the presence of specific growth factors were not explored. For example, HTF was used as the insemination medium, which lacks amino acids. The temporary deprivation of amino acids during this critical fertilization period may have contributed to some of the downstream metabolic and proteomic differences observed in blastocysts, independent of oxygen tension or culture conditions. Therefore, while our findings highlight significant changes induced by embryo culture, it is possible that some differences, including those observed under 5% oxygen, are also influenced by the IVF process itself. Also, one potential limitation of the study is that the oxygen concentration during the fertilization stage was kept at 5%. This may have led to subtle metabolic or proteomic differences at the fertilization stage, which could not be fully accounted for in our analysis of the post-fertilization stages. In addition, we only used KSOM medium. This choice was made because it was found to be: (i) optimal for mouse preimplantation embryo culture (Perin *et al.*, 2008); (ii) the basis for the commercially available Global medium, used widely in human IVF (Wiemer *et al.*, 2002); and (iii) we (Rinaudo and Schultz, 2004; Bloise *et al.*, 2012) and others (Ecker *et al.*, 2004; Bolton *et al.*, 2016; Vrooman *et al.*, 2022) have generated extensive physiological, transcriptomic, and metabolic data with this medium, such that the results of the current study can be connected to and integrated with the previous analyses. One potential limitation of KSOM is its inclusion of L-glutamine. Although L-glutamine is essential for cellular metabolism, its breakdown during *in vitro* culture can lead to ammonium production, a byproduct known to be toxic to developing embryos (Wale and Gardner, 2013). The combination of high oxygen concentrations, which promote ROS formation, and ammonium accumulation from glutamine breakdown may exacerbate cellular stress in developing embryos (Wale and Gardner, 2013), potentially disrupting the delicate balance required for proper embryonic development.

Therefore, our findings should be interpreted within the context of this specific medium. Future studies incorporating different culture media will be essential to determine whether the observed metabolic and proteomic changes are consistent across various embryo culture conditions.

Moreover, while we focused on the metabolic and proteomic profiles at the blastocyst stage, further studies examining earlier stages of development, such as the zygote or morula, may provide additional insights into the early responses of embryos to culture conditions. Another limitation is that we investigated only the metabolic and proteomic signature of embryos generated by conventional IVF. Similarly to our findings on the transcriptome of IVF- or ICSI-generated blastocysts (Giritharan *et al.*, 2010), it is likely that embryos generated by ICSI will have a significantly different profile. Therefore, the applicability of our findings to ICSI-derived embryos remains uncertain, and further studies comparing the two approaches are warranted. Also, we acknowledge that the relatively small sample size used in our IF assays may limit the statistical power and generalizability of these findings. Future studies with larger sample sizes are warranted to validate our observations and strengthen the reliability of the IF results.

In summary, these findings offer valuable insights into the significant impact of IVF culture conditions, particularly oxygen levels, on the metabolic and proteomic landscapes of embryos. By identifying key metabolic pathways affected by oxidative stress, this study sheds light on potential molecular mechanisms driving metabolic adaptation during early embryonic development. We have emphasized that the integration of these omics technologies not only sheds light on how oxidative stress affects cellular function but also provides a deeper understanding of how early metabolic stress may influence long-term health outcomes, as proposed by the developmental origin of health and disease (Fleming *et al.*, 2018). These insights have direct clinical relevance, laying the groundwork for optimizing ART protocols to minimize oxidative stress and improve embryo quality. By emphasizing the value of proteomics and metabolomics in understanding the molecular mechanisms of embryonic development under various IVF conditions, we believe our study provides a crucial step toward improving ART outcomes and mitigating oxidative stress-related risks.

In conclusion, our study provides a comprehensive analysis of the proteomic and metabolomic landscape of IVF-generated embryos, shedding light on the intricate metabolic dynamics accompanying early embryonic development. This knowledge is crucial for optimizing culture conditions, ART procedures, and ultimately enhancing the long-term health outcomes of individuals conceived through ART.

## Supplementary Material

hoaf022_Supplementary_Data

## Data Availability

All data generated or analyzed during this study are included in the manuscript and supporting files. Proteomic and metabolomic data are available upon request.
