# Supplementary material for: Proteomic and metabolomic insights into oxidative stress response activation in mouse embryos generated by in vitro fertilization
Source: Hum Reprod Open. 2025 Apr 28;2025(2):hoaf022. doi: 10.1093/hropen/hoaf022 (PMC12101870; doi:10.1093/hropen/hoaf022)
Supplement: hoaf022_Supplementary_Data [file hoaf022_supplementary_data.zip › Supplementary_File_Legends_041525.docx]

**Title**: Proteomic and Metabolomic Insights into Oxidative Stress Response Activation in Mouse Embryos Generated by In Vitro Fertilization

**Authors**: Seok Hee Lee, Saúl Lira-Albarrán, Paolo F. Rinaudo

**Supplementary File S1.** Proteomic analysis data for IVF5%O₂ versus FB. This supplementary file contains detailed results of the proteomic analysis comparing protein expression profiles between IVF5%O₂ and FB conditions. The data include quantitative measures of protein levels, identification of significantly altered proteins, and their associated pathways. (A) List of 426 differentially expressed proteins (|log₂FC| > 1, adjusted p-value < 0.05). (B) Enriched canonical pathways defined by KEGG. (C) Overrepresented parental biological processes. (D) Overrepresented parental molecular functions. (E) Enriched canonical pathways defined by Reactome. (F) Enriched canonical pathways defined by WikiPathways.

**Supplementary File S2**. Proteomic analysis data for IVF20%O₂ versus FB. This file contains results of proteomic comparisons between IVF20%O₂ and FB groups. (A) List of 599 differentially expressed proteins (|log₂FC| > 1, adjusted p-value < 0.05). (B) KEGG pathway enrichment. (C) Overrepresented biological processes. (D) Overrepresented molecular functions. (E) Reactome pathway enrichment. (F) WikiPathways enrichment.

**Supplementary File S3**. Proteomic analysis data for IVF5%O₂ versus IVF20%O₂. The data include identification of significantly altered proteins and their associated pathways. (A) List of 244 differentially expressed proteins (|log₂FC| > 1, adjusted p-value < 0.05). (B) KEGG pathway analysis. (C) WikiPathways enrichment.

**Supplementary File S4**. Metabolomic analysis data for IVF5%O₂ versus FB. This file contains quantitative metabolite measurements and pathway enrichment data. (A) List of 82 metabolites (confidence levels 1–3, p-value < 0.05). (B) Enriched metabolite sets.

**Supplementary File S5**. Metabolomic analysis data for IVF20%O₂ versus FB. (A) List of 75 metabolites (confidence levels 1–3, p-value < 0.05). (B) Enriched metabolite sets.

**Supplementary File S6**. Metabolomic analysis data for IVF5%O₂ versus IVF20%O₂. This file contains comparative metabolomics data between IVF5%O₂ and IVF20%O₂ conditions. (A) List of 77 metabolites (confidence levels 1–3, p-value < 0.05). (B) Enriched metabolite sets and related metabolic pathways.

Note: These supplementary files have been provided separately.
