# Supplementary figures and images for: Proteomic and metabolomic insights into oxidative stress response activation in mouse embryos generated by in vitro fertilization
Source: Hum Reprod Open. 2025 Apr 28;2025(2):hoaf022. doi: 10.1093/hropen/hoaf022 (PMC12101870; doi:10.1093/hropen/hoaf022)

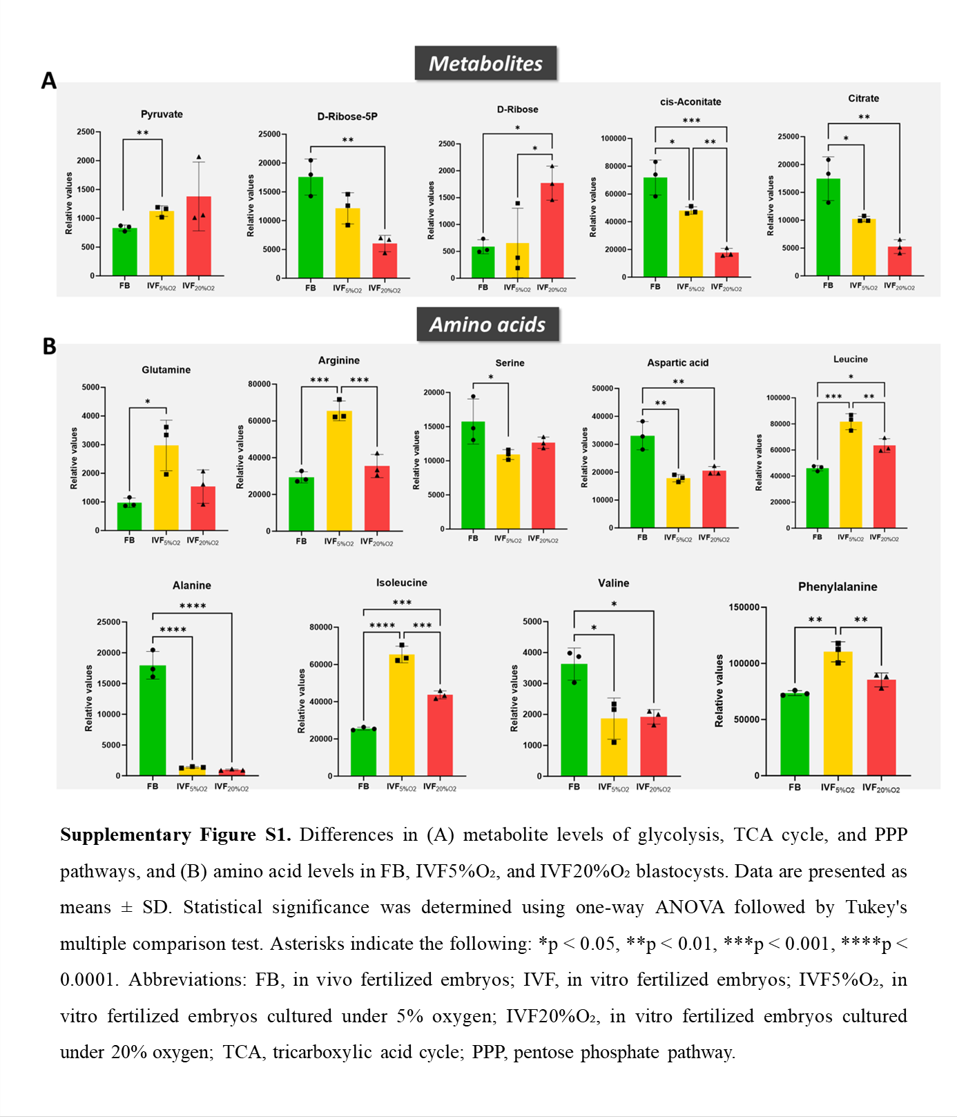

Supplement: hoaf022_Supplementary_Data [file hoaf022_supplementary_data.zip › Supplementary_figure_1_metabolite&amino_acids_032425.tif]

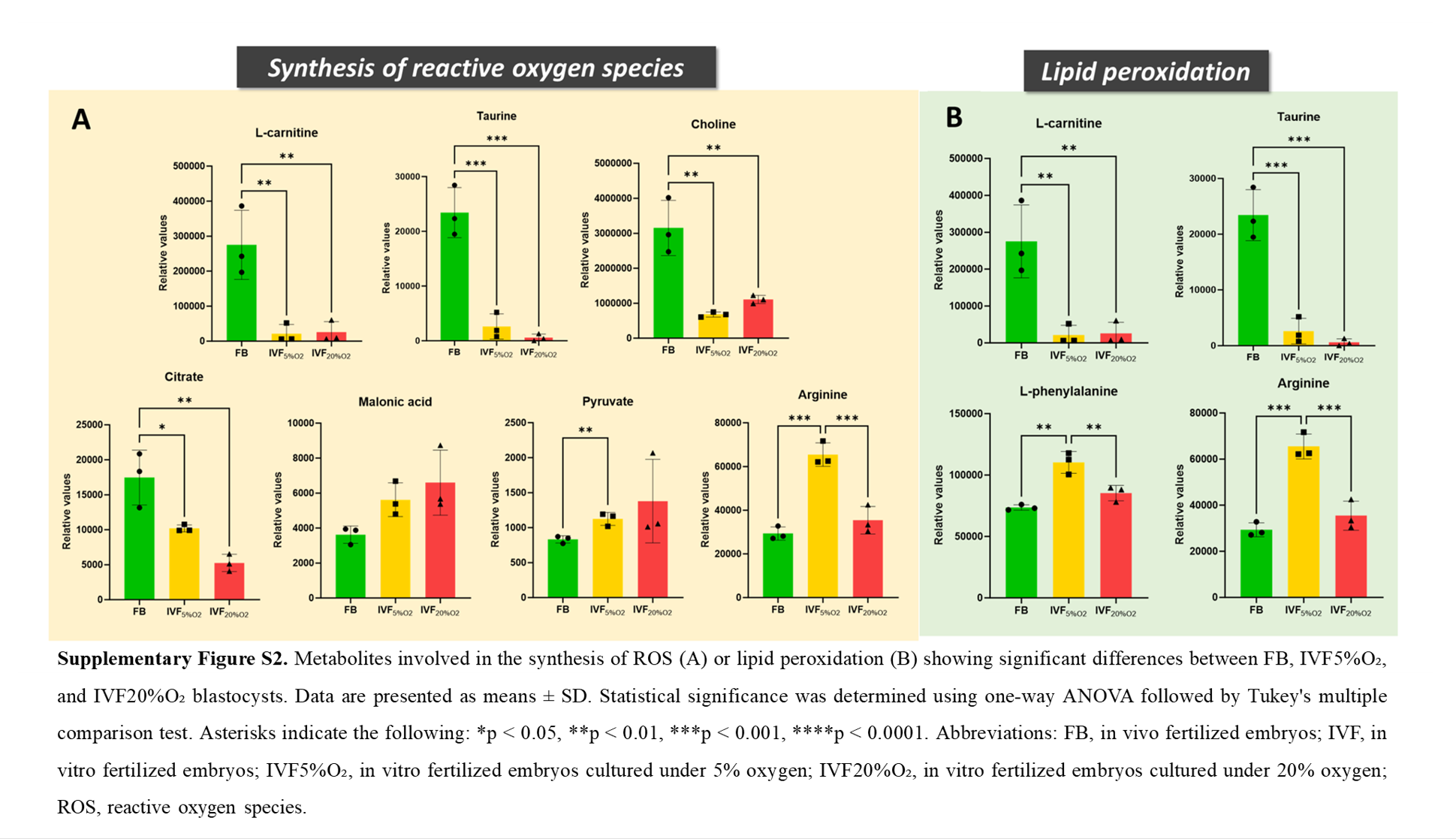

Supplement: hoaf022_Supplementary_Data [file hoaf022_supplementary_data.zip › Supplementary_figure_2_ROS&lipid_peroxidation_032425.tif]
